# The impact of lockdown on young people with genetic neurodevelopmental disabilities: a study with the international participatory database GenIDA

**DOI:** 10.1186/s12888-022-04213-6

**Published:** 2022-08-25

**Authors:** Romain Coutelle, Morgane Boedec, Karlijn Vermeulen, Joost Kummeling, David A. Koolen, Tjitske Kleefstra, Camille Fournier, Florent Colin, Axelle Strehle, David Geneviève, Pauline Burger, Jean-Louis Mandel

**Affiliations:** 1 Child and Adolescent Psychiatry Service - Department of Psychiatry, University Hospitals of Strasbourg, Strasbourg, France; 2 Department of Psychiatry, INSERM1114, University Hospitals of Strasbourg, University of Strasbourg, 1 place de l’hôpital, 67000 Strasbourg, France; 3 Department of Human Genetics, Donders Institute for Brain, Cognition and Behaviour, Radboud University Medical Center, Nijmegen, The Netherlands; 4 Karakter Child and Adolescent Psychiatry, Department of Intellectual Disabilities, Ede, The Netherlands; 5 Centre of Excellence for Neuropsychiatry, Vincent Van Gogh Institute for Psychiatry, Venray, The Netherlands; 6 Institute of Genetics and Molecular and Cellular Biology (IGBMC), Dept. of Neurogenetics and Translational Medicine), Illkirch, Strasbourg, France; 7 Institut National de La Santé Et de La Recherche Médicale, U 1258, Illkirch, 67400 Strasbourg, France; 8 Centre National de La Recherche Scientifique, UMR 7104, Illkirch, 67400 Strasbourg, France; 9 University of Strasbourg, Strasbourg, France; 10 Current Address: INSERM UMR_S1109, Tumor Biomechanics Lab, Fédération de Médecine Translationnelle de Strasbourg (FMTS), University of Strasbourg, Strasbourg, France; 11 Département de Génétique Maladies Rares, University of Montpellier, CLAD Sud Languedoc-Roussillon, INSERM U1186, Montpellier, France; 12 Institute for Advanced Studies, University of Strasbourg (USIAS), Strasbourg, France

**Keywords:** Intellectual disability, Autism spectrum disorder, Behavioural problems, Genetic disorders, COVID-19, Self-report, Participatory online survey

## Abstract

**Background:**

Previous publications suggested that lockdown is likely to impact daily living issues of individuals with intellectual disabilities. The authors notably suspected an intensification of behavioural, eating and sleep problems.

**Methods:**

To test these hypotheses, we conducted an international online survey about the impact of COVID-19-associated first lockdown on people with genetic neurodevelopmental disorders. This survey was carried out using GenIDA, an international participatory database collecting medical information on genetic neurodevelopmental disorders. Patients’ relatives took part in this online survey from 30/04/2020 to 09/06/2020. This survey adapted from GenIDA standard questionnaire requested information on diagnosis, lifestyle and was based on yes/no answers to questions regarding behaviour, diet, and sleep, in the 6-months period before lockdown and during lockdown. We also asked relatives to evaluate the intensity of these problems by severity level. Finally, relatives could freely comment in open fields on the medical and/or quality of life problems they had encountered during lockdown.

**Results:**

In total 199 participants—144 children and 45 adults—with neurodevelopmental disorders (intellectual disability (79.4%) and/or autism spectrum disorder (21.6%)) of various genetic origins, with near-equal male/female (96/103) contribution and originating mainly from Europe and Northern America, were included. The average lockdown duration at time of the survey was 57 days. We did not find differences in the frequency of behavioural, eating and sleep problems before and during lockdown. Moreover, there was no apparent difference in the intensity of eating and sleep disorders between both periods. However, for persons with behavioural problems at both periods, relatives reported an increase in aggressivity, self-aggressivity, depressiveness, stereotypies, and restricted interests during lockdown, all of which might be interpreted as consequences of a lack of stimulation or a reaction to unexpected changes in daily habits.

**Conclusions:**

Our results support previous studies that suggest that the negative impact of lockdown does not depend on the intellectual disability per se but on the associated comorbidities such as behavioural disorders. This study addresses the need for prevention of behavioural disturbance in the vulnerable population with genetic neurodevelopmental disabilities.

**Supplementary Information:**

The online version contains supplementary material available at 10.1186/s12888-022-04213-6.

## Background

### The impact of COVID-19 on people with intellectual disabilities

Early publications on COVID-19 highlighted the impact that this disease might have on people with intellectual disabilities (ID) [1–3]. These individuals may be at greater risk of infection for several reasons that include physical health problems and social circumstances. Previous studies have evidenced that people with ID have higher prevalence of specific comorbidities, such as hypertension, heart- or respiratory diseases, and diabetes [1, 2]. All these disorders are known risk factors for poor outcomes in COVID-19 patients [1–3]. Moreover, cognitive impairments can limit the understanding of prevention measures [1–3]. Epidemiological studies of COVID-19 outcomes in New York (USA) have confirmed the initial assumption that this disease provides a higher risk for people with intellectual and developmental disabilities, particularly young people [2] and people living in community housing [4]. However, the impact of COVID-19 in people with ID is not limited to this higher risk of infectious diseases [5] but also involves the day-to-day consequences of the measures to control the spread of COVID-19.

### The impact of COVID-19-related restrictions and lockdown on the mental health of people with ID

In response to the rapid spread of Covid-19 around the world, many governments have implemented international travel restrictions and national lockdowns between March and May 2020 (dates vary somewhat by country) to limit travel within their borders and prevent people from mixing in public. In France, the US, the UK, and the Netherlands notably, the first Covid-19 containment was characterized by stay-at-home orders, meaning that people were asked to work from home whenever possible, and to leave their homes only for essential reasons (to shop for food and medicine, to engage in one physical activity per day, to care for a vulnerable person, or for medical reasons). All non-essential business premises, such as restaurants, were closed. Schools were closed to all students, except for the children of key workers. It was expected that the COVID-19-linked constraints such as visitor restrictions and lockdown would impact mental health in individuals with ID [6]. The misunderstanding of its importance and the implications of not adhering to restrictions might even compound these difficulties. A Spanish online survey completed by 582 participants with intellectual and developmental disabilities confirmed that people under 21 years of age (38.6%) and students (27.2%) found it difficult to understand information about COVID-19 [7]. Moreover, people with ID often follow their own routines and need to be prepared for changes. If not, sudden changes can increase their level of anxiety causing behavioural challenges and potentially mental health conditions [1, 8]. Depression and anxiety might also be observed in such exceptional circumstances [1, 9, 10]. A Polish study based on 64 telephone interviews confirmed this initial hypothesis by showing that more than one third of the participants with developmental disabilities reported mild or more severe symptoms of anxiety and depression [11]. Another study of adults with Down Syndrome evidenced an exacerbation of depressive symptoms and a deterioration in functional status [12].

### Risk factors of lockdown on the mental health of people with ID

Highly prevalent comorbidities in ID such as Autism Spectrum Disorder (ASD) [13] or Obsessive Compulsive Disorder (OCD) [14] might be hampering mental health in case of lockdown. Individuals with ID and ASD might be overwhelmed by the information related to COVID-19 giving rise to an invasive restricted interest [6, 8]. Due to the properties of restricted interests, when people with ASD become interested in a topic, they are likely to engage with it intensively and with a strong impact on their daily lives [15, 16]. These individuals might also be stressed by changing daily routines which is particularly difficult in ASD because of the resistance to change associated with this disorder [6–8]. Because of comorbid OCD in ID, the need for scrupulous personal hygiene might also trigger obsessive thinking about cleanliness and compulsions about hand washing [1, 6, 9].

Another risk is a possible aggravation of the patient’s symptomatology due to the cessation of medical follow-up [1, 8]. Thus, more requests for psychotropic medication are expected to arise, in an attempt to support people with ID and to assist families and caregivers in managing behavioural problems [6]. During the lockdown period, total visits in the community ID service in two English districts increased by 19 per week and medication interventions increased by two per week [17]. Hypnotics and benzodiazepines were the most frequently prescribed psychotropic medications during the lockdown period [17].

Beyond behavioural problems, sleep and eating disorders that are frequently reported in patients with neurodevelopmental disorders (NDDs) may also potentially worsen during lockdown, as sleep disorders in both children and adults in the general population clearly increased during lockdown [18, 19]. Information on sleep and eating disorders in people with ID during lockdown is limited. However, one study in 20 children with learning disorders and comorbidities (such as ASD, Attention Deficit Hyperactivity Disorder (ADHD), epilepsy, behavioural disorders, language disorder or childhood psychosis) undertaken in Cordoba [20] showed that sleep disorders, anxiety and eating disorders prevailed during the lockdown.

An online study of 38 Italian children with Fragile X syndrome evidenced a general deterioration in sleep quality (difficulty falling asleep with an increase in the time to fall asleep and the frequency of night awakenings) and an increase in behavioural problems [21]. Using an online questionnaire, Wieting et al*.*’s results suggest that the COVID-19 pandemic affects the mental health of individuals with Prader-Willi syndrome (PWS), resulting in an increase in behaviours associated with PWS, including food-seeking, temper outbursts, and irritability [22]. These results contrast with those of a French study that associated lockdown with positive effects for most adults with PWS studied [23]. However, the two studies differ widely: the respondents in the German study were parents of children with PWS, whereas the French study relied on self-reports completed by adults with PWS.

### Hypotheses on the impact of the lockdown on mental health of people with ID and/or other NDDs

Previous studies [1–3, 6] emphasized the possible impact of the COVID-19-related lockdown on daily living issues in people with ID. Based on these and their clinical experience, the authors hypothesized that individuals with genetic NDDs may be more prone to develop behavioural, sleep and eating problems during lockdown. This was investigated for the first lockdown period in spring 2020 by creating an ad hoc questionnaire and informing the GenIDA participating relatives of the study. We had 199 relatives completing the specific online questionnaire on behalf of an individual (children or adult) affected by a genetically determined NDD.

## Methods

### Issues in studying the lockdown’s impact on mental health of people with ID and/or other neurodevelopmental disorders and the GenIDA database

The study of lockdown’s impact required rapid implementation, easy access to information about persons with neurodevelopmental disorders (NDDs) and major involvement of relatives; the GenIDA database displays all these features. GenIDA [24] is an international online participatory database for patients with genetic forms of intellectual disability with or without autism or epilepsy. It aims to collect medically relevant information on the manifestations and the natural history of these issues, useful for families and professionals to improve care and treatment of individuals with such genetic NDDs. GenIDA is dedicated to persons with genetic NDDs, a well-defined group within the heterogeneous ID population, as well as to doctors, researchers and other professionals involved in the management and the care of these conditions [25]. Since its online launch by the end of 2016, the GenIDA project received the support of many professionals and patients’ associations for the recruitment of patients, and currently, the database encompasses 1450 participants having filled on average 90% or more of the questionnaire. The relevant information (natural history, medical comorbidities, cognitive and behavioural aspects, including sleep and eating disorders, response to treatments, etc.) is entered in the database and updated by the relatives of the affected individual, based on a structured questionnaire (41 multiple choice questions and 5 open text questions) currently available in 7 languages [24]. A dedicated control-access system was designed [26] to fine-tune the access of families to disease-specific questionnaires and to specific aggregated data analyses. Registered professionals have access to such de-identified statistical data. GenIDA’s International Scientific Advisory Board (consisting in professionals—clinicians, researchers, etc.—as well as representatives of patients’ associations) meets annually. The GenIDA project was notably able to heavily recruit participants with Koolen-de Vries syndrome (KdVS—17q21.31 deletion / *KANSL1* mutation) and to use this cohort as proof of concept of the value of such a participatory approach [27].

### Participants

The online survey (in English and French) was implemented in the GenIDA database in spring 2020 during the first wave of lockdowns worldwide (i.e., between March and May / June 2020). All the registered GenIDA participants were contacted by e-mail about this study: the message specified the main objective of the study and the inclusion criteria, namely that participants had to be registered in GenIDA and undergo lockdown. Information was also spread through the social medium Facebook and via patient’s associations. Then, patients’ relatives completed this survey from 30/04/2020 to 09/06/2020. No selection of participants was made on the basis of language; they simply had to understand the survey in order to complete it and some families used an online translator to participate. In addition, participants could answer the open-ended questions in their own language. RC, JLM and AS selected two questions ["What are the main problems that affected the daily life (quality of life) of the person concerned UNDER confinement/confinement?" and "What are the main medical problems that occurred UNDER confinement/confinement?"], which focus on the main problems, to be relevant and very open-ended, and thus be as inclusive as possible (Additional file 1). Among the 199 participants selected for this study, we recorded responses in English and French, but also in Dutch, German, and Italian.

### Questionnaire

This survey requested medical information and consisted of questions regarding behaviour, diet, and sleep 6 months before and during lockdown. The inquiry began with actualised information about diagnoses. Relatives completed patient’s diagnosis status about ID and ASD. When available, they also provided the score(s) from any test(s) performed to assess ID and ASD. Lifestyle information (such as living with or outside the family, sharing a bedroom or having one’s own bedroom, living in a house or apartment, and having easy access to a park or garden) was requested. In addition, relatives answered “yes”/ “no” to questions about the affected individual’s behavioural issues, as well as eating and sleep problems 6 months before and during lockdown. The answer “no” corresponded either to “no problem” or “I don’t know”. We acknowledge that a “no” response may differ from “I don’t know”. However, the proportion of the latter response was limited. The answer "I don't know" was selected in 2%, 0.5% and 2.5% of the cases respectively to the questions concerning behavioural, eating and sleeping problems before lockdown. Relatives also had to assess the intensity of these problems during these two periods exclusively in case of a positive answer to these questions. Thus, when relatives answered “yes” to the question concerning behavioural problems, a list of 13 types of problematic behaviours appeared: aggressivity; self-aggressivity (self-mutilation); impulsivity; hyperactive; attention deficit; shy; anxious; depressive tendencies; restricted interests; repetitive behaviour / stereotypes; obsessions; phobias; problems related to a former diagnosis of schizophrenia. When sleep problems were reported, relatives were also asked to indicate whether they were perceived as mild, moderate, or major. Similarly, when eating problems were reported, a list of four descriptors (eats too much / craves for food / eats only very restricted food / does not want to eat) was proposed to describe them more precisely, and relatives could further complete their answer using free text. Relatives also had to assess the perceived severity of eating problems (mild, moderate, major). Sociability towards familiar and unfamiliar adults, as well as towards familiar and unfamiliar children had to be assessed as well using the following descriptors: very sociable, average sociability, little sociability or no interaction. Finally, relatives could comment their answers regarding medical and quality of life problems during lockdown in open text.

We established scores to assess the severity of behavioural, sleep and eating problems. Each type of behavioural problem was rated 1, 2, 3 or 4 on a four-point Likert scale depending on whether the response was “non-existent”, “mild”, “moderate”, or “major” respectively. The overall behaviour problems were thus rated from a minimum of 13 to a maximum of 52. Similarly, eating and sleep problems were evaluated on a three-point Likert scale ranging from 1 to 3 according to the degree of severity reported, i.e., “mild”, “moderate” or “major”. Finally, a four-point Likert scale from 1 to 4 was used to measure the level of sociability ranging from “no interaction” to “very sociable” for each type of social interaction, i.e., “familiar adults”, “unfamiliar adults”, “familiar children” and “unfamiliar children”. The overall “sociability” was thus rated from 4 to 16.

## Analysis

All comparisons of nominal data between the two periods, i.e., before and during the lockdown (“yes”/ “no” responses) were carried out using the McNemar test for paired samples. Quantitative variables corresponding to the intensity of behavioural, eating and sleep problems before and during the lockdown were compared using Student’s t-test for paired samples. When variables did not satisfy normality assumption, Wilcoxon signed-rank test was used. In case of missing values, we excluded cases analysis by analysis.

We also carried out repeated measures ANOVA using the intensity of behavioural, eating or sleep problems before and during lockdown as explained variables, and age, gender, severity of ID, ASD diagnosis (presence/absence), housing characteristics (such as living with or outside the family, sharing a bedroom or having one’s own bedroom, living in a house or apartment, and having easy access to a park or garden) as explanatory variables. The same test was also carried out using sociability before and during lockdown as an explained variable. A statistical threshold was set at *p* < 0.05.

With regard to the qualitative analysis related to quality of life issues, RC and PB checked and read all responses, and identified two main themes regarding their positive, negative, or neutral valence on the one hand and the consequences of stopping therapy and/or intervention during lockdown on the other. Positive valences refer to the perception of a better quality of life during lockdown (e.g. good relational issues, less problematic behaviour, positive emotions in people with ID). Comments indicating "no change" or "no problems" were also considered positive valence. Negative valences were related to worsening behaviour during lockdown or to daily problems (e.g. more attention needs). Neutral valences referred to a mix of positive and negative statements during lockdown. Each open-ended comment was appraised by two raters: the inter-rater reliability for valences measured by Cohen’s Kappa was 0.85. The inter-rater reliability for the consequences of stopping therapy and/or intervention during lockdown was 0.75.

For the qualitative analysis related to medical problems, RC and PB checked and read all responses, and identified all the relevant medical domains (e.g. digestive problems, mental health problems).

We did not use any software to analyse the content of the responses., However, we use the free version of the DeepL software [28] for translation when necessary.

## Results

We collected 218 responses to questionnaires (Additional file 1). Those without any information about behavioural, eating or sleep problems during lockdown were excluded. 199 children, adolescents, and adults with a combined total of 34 different types of genetic defects (Fig S1) living in 16 different countries (Fig S2) were included.

In almost 67% of cases, the mother responded to the lockdown-related questionnaire. Both parents responded in 18% of cases, and the father alone responded in 6.5% of cases. The person with ID her/himself answered the questionnaire in 2.5% of the cases. In the other cases, it was one of the grandparents, or the siblings, and sometimes even a referent/guardian who responded to the survey.

The characteristics of the population included are presented in Table 1. The age of the participants ranged from 2 to 61 years, with an average age of 14.0 years, and male/female contribution was roughly equal (96/103). The frequencies of ID and ASD were 79.4% and 21.6% respectively. The average of available IQ values was 53.9 (*n* = 53). Half of the ASD diagnoses (53.3%) were established in expert centres using standard tests such as the ADI-R [29] or the ADOS [30].

**Table 1:** Clinical characteristics of participants (nominal variables)

	**Participants (*n* = 199)**	
	***n***	**%**
**Children / Adults**	154 / 45	77.6 / 22.6
**Gender: Male / Female**	96 / 103	48.2 / 51.8
**Diagnosis of Intellectual Disability (ID)**	158	79.4
Mild Intellectual Disability	26	13.1
Moderate Intellectual Disability	72	36.2
Severe Intellectual Disability	49	24.6
Profound Intellectual Disability	11	5.5
**Diagnosis of Autism Spectrum Disorder (ASD)**	43	21.6
**Diagnosis of ASD and ID Housing characteristics during lockdown**	33	16.6
The affected individual lives within the family	181	91.0
The affected individual lives in a family home (not in an apartment or other type of housing)	148	74.3
The affected individual has his/her own bedroom	159	79.9
The affected individual has an easy access to a garden or park	159	79.9

91% of the respondents indicated that the person with ID for whom they were responding to the survey was living with them within the family during the lockdown (8% indicated that the person with ID lived outside the family and only 1% did not reply to that question). Among them, 75% lived in a single-family home, and 20% in an apartment. Some respondents mentioned (in free text) that person with ID lived in a foster home, sometimes medicalized, in a centre for the disabled, in a specialised reception centre (home for male adults, centre specialised for the mentally ill) or was supported by carers in his/her own home.

We did not find any difference on the frequency of behavioural, eating and sleep problems before and during lockdown (Table 2).

**Table 2:** Behavioural, eating and sleep problems in affected individuals before and during lockdown (nominal variables)

	**Affected individuals before lockdown (*n* = 199)**		**Affected individuals during lockdown (*n* = 199)**		***p-*value**
	*n*	%	*n*	%	
Behavioural problems	106	53.3	107	53.8	0.77
Eating problems	62	31.2	59	29.6	1
Sleep Problems	69	34.7	67	33.7	1

**Fig. 1:**
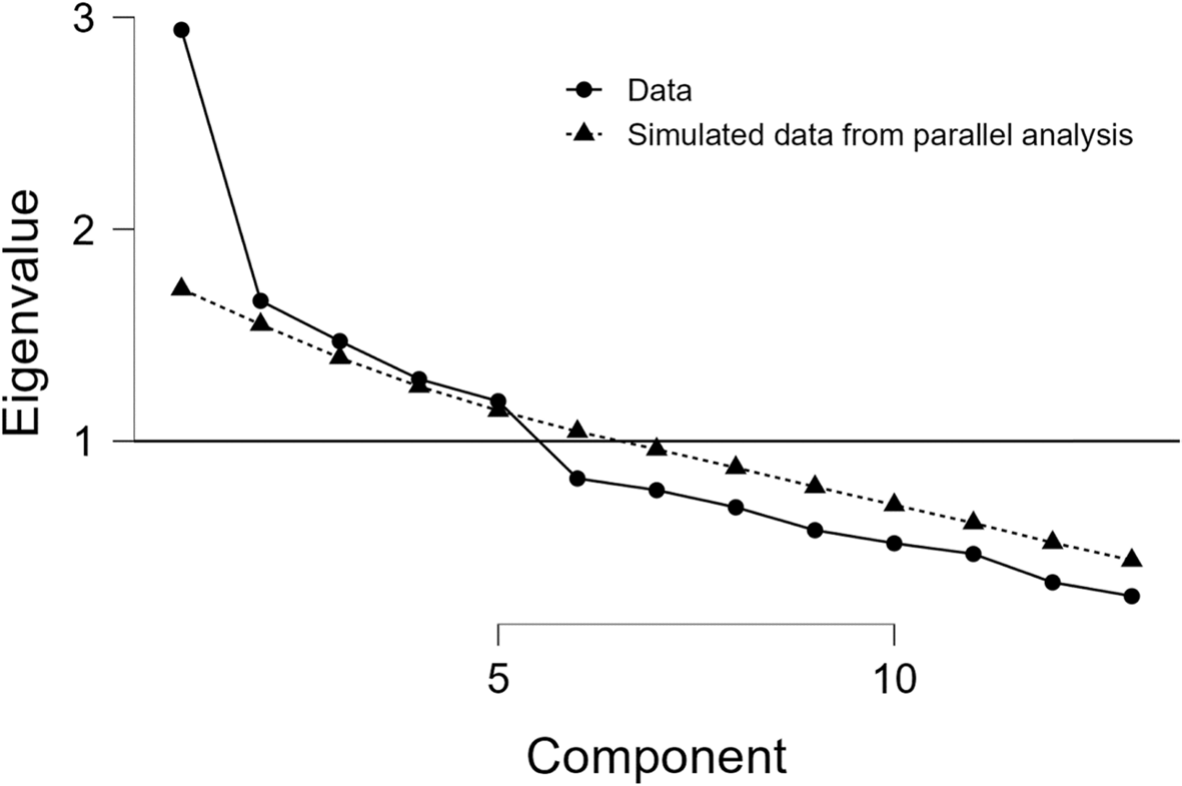
Scree plot with Eigenvalue and components of problematic behaviours

As explained in the Analysis section above, we assessed 13 types of problematic behaviours using a four-point Likert scale and a total score that was a sum of the scores for these 13 types of behaviours. To investigate the internal consistency of this composite score, we performed a principal component analysis (PCA) with the values related to the lockdown period. The eigenvalues are presented in the scree plot (Fig. 1).

This diagram clearly showed one main dimension and supported the one-dimensional nature of the measure. The best fit of the model was based on five classic psychiatric factors that referred to internalizing problems (depression, phobia, anxiety, shyness and obsession), externalizing problems (attention deficit, hyperactivity and impulsivity), aggressivity (self- and hetero-aggressivity), autistic behaviours (restricted interests and repetitive behaviours) and schizophrenia diagnosis (Table 3). The variable related to schizophrenia diagnosis was alone.

**Table 3:** Component loadings of problematic behaviours during lockdown

Type of problematic behaviour	Internalizing problems	Autistic behaviours	Agressivity	Externalizing problems	Schizophrenia diagnosis
Anxiety	0.819				
Depressivity	0.750				
Phobia	0.721				
Obessions	0.571				
Shyness	0.429				
Restricted interests		0.880			
Repetitive behaviour, stereotypes		0.815			
Hetero-agressivity			0.770		
Auto-agressivity			0.637		
Impulsivity				0.523	
Hyperactivity				0.768	
Atttention deficit				0.682	
Shizophrenia diagnosis					0.819

*Note:* Applied rotation method is promax

Finally, we examined the reliability of all the behavioural problem variables. Cronbach's alpha was 0.69 and was therefore considered satisfactory.

A difference in intensity of the reported problems was calculated for the subjects, who experienced behavioural problems before as well during lockdown (*n* = 84). During lockdown the reported intensity of behavioural problems was higher for the following categories: aggressivity, self-aggressivity, depressiveness, stereotypies and restricted interests (Table 4). In contrast to this increase in negative behaviours, the affected individuals were also generally less shy during lockdown than before. Intensity of behavioural problems before and during lockdown does not appear to interact with age, gender, intensity of ID, ASD diagnosis (presence/absence) or housing characteristics (Table S1). No differences in the intensity of eating (*n* = 51; *p* = 1) and sleep problems (*n* = 57; *p* = 0.096) were evidenced between both periods. Neither age, gender, intensity of ID, ASD diagnosis (presence/absence), nor housing characteristics interact with intensity of eating and sleep problems before and during lockdown.

Except with familiar adults, there was less sociability during lockdown. Relatives reported that the affected individuals had fewer social interaction during lockdown with unfamiliar adults, and with both familiar and unfamiliar children (Table 5). However, social relationships with familiar adults have been spared during this period. The intensity of behavioural problems before and during lockdown was not associated with any of the factors studied (Table S2).

**Table 4:** Intensity of behavioural, eating and sleep problems before and during lockdown (quantitative variables)

	Intensity of behavioural problems before lockdown ^*^	Intensity of behavioural problems during lockdown ^*^	**Cohen’s *d***	**Rank-Biserial Correlation**	**95% confidence interval**		***p*-value**
	**Mean (SD)**	**Mean (SD)**			**Lower**	**Upper**	
**Category of behavioural problems**							
Aggressivity	1.82 (0.79)	2.17 (0.92)		-0.82	-0.92	-0.63	< 0.001 ^**, a^
Self-aggressivity	1.81 (0.86)	1.94 (0.94)		-0.65	-0.87	-0.22	0.008 ^**, a^
Obsessions	2.44 (0.99)	2.56 (1.12)		-0.27	-0.59	0.12	0.131 ^a^
Phobias	1.75 (0.84)	1.65 (0.91)		0.23	-0.24	0.61	0.330 ^a^
Problems related to a former diagnosis of schizophrenia	1.07 (0.30)	1.01 (0.18)		1.00	1.00	1.00	0.149 ^a^
Impulsivity	2.85 (0.94)	3.01 (0.97)		-0.33	-0.64	0.07	0.079 ^a^
Hyperactivity	2.25 (1.16)	2.31 (1.16)		-0.29	-0.69	0.25	0.263 ^a^
Attention Deficit	3.21 (0.84)	3.24 (0.88)		-0.15	-0.54	0.29	0.459 ^a^
Shyness	1.77 (0.80)	1.51 (0.72)		0.64	0.29	0.84	0.004 ^**, a^
Anxiousness	2.53 (0.96)	2.67 (1.07)		-0.27	-0.61	0.15	0.187 ^a^
Depressive tendencies	1.37 (0.68)	1.55 (0.93)		-0.57	-0.81	-0.15	0.013 ^**, a^
Restricted interests	2.31 (1.03)	2.67 (1.06)		-0.83	-0.93	-0.62	< 0.001 ^**, a^
Repetitive behaviour / stereotypes	2.76 (0.93)	3.05 (0.97)		-0.74	-0.88	-0.49	< 0.001 ^**, a^
Total score	27.53 (4.45)	28.93 (5.58)	-0.38		-0.63	-0.13	0.003 ^**, b^

^*^ Comparisons were limited to affected individuals who showed behavioural problems at both periods (see main text for detailed explanation)

^**^ Significant at *p* < 0.05

^*a*^ Wilcoxon signed rank test

^*b*^ Student’s t-test for paired samples

Open-ended questions referred first to “quality of life” problems and then to medical problems during lockdown. Relatives did not provide comments in 8% of cases. The valence of the comments was positive, negative or neutral in 13%, 71% and 9% of the cases, respectively. Some examples are displayed in Table 6, part A. The consequences of therapy and/or interventions cessation during lockdown were also rated by two evaluators. These consequences were mentioned in 28% of cases. Some examples are displayed in Table 6, part B.

**Table 5:** Evolution of sociability in affected individuals before and during lockdown (quantitative variables)

	Sociability before lockdown	Sociability during lockdown	Rank-Biserial Correlation	95% confidence interval for Rank-Biserial Correlation			*p*-value
	Mean (SD)	Mean (SD)		Lower	Upper		
Interaction with							
familiar adults	3.77 (0.51)	3.77 (0.49)	0.00	-0.55		0.55	1.000 ^a^
unfamiliar adults	2.88 (0.85)	2.38 (1.12)	0.92	0.84		0.96	< 0.001 ^*, a^
familiar children	3.32 (0.82)	3.02 (1.15)	0.57	0.28		0.77	0.001^*,a^
unfamiliar children	2.55 (1.08)	2.08 (1.13)	0.87	0.75		0.94	< 0.001 ^*, a^
Total score	11.62 (4.35)	9.55 (4.91)	0.65	0.46		0.78	< 0.001^*, a^

^*^ Significant at *p* < 0.05

^*a*^ Wilcoxon signed rank test

**Table 6:** Selected examples of relatives’ comments reported in open-ended questions in GenIDA

**A**	**General comments regarding the lockdown situation**	**Perception**
	“She is normally insanely social, and the restrictions are very hard for her. She is anxious if people don’t text back right away or wants to FaceTime—she’s convinced they’re mad at her. This happens during normal life too, but it has gotten worse. She and her sister are also bickering more than usual. She is immensely apologetic for every single thing. She wants to call people and FaceTime with anyone she can.”	Negative
	“He ruminates on news headlines—retelling the same info over and over. He gets frustrated easily, will talk disrespectfully and even physically push his sisters if they disagree with him on something.”	Negative
	“My son has little change in behaviour during this confinement. We live in the country, so during a regular summer, we wouldn't have much interaction with others anyway. The main challenge has been for us as adults to recreate a type of school schedule so he can continue his education from home.”	Neutral
	“We are having a great time as a family, there is a very good atmosphere in the family and the stress of everyday life has disappeared (especially the departure times). I think this has calmed our son down a lot. Thanks to him, we go for long walks (in the mountains without meeting anyone), we have great exchanges with him, we play, and this is good for everyone, including him.” [translated from French]	Positive
**B**	**Relative’s opinion about the consequences of therapy or education cessation during lockdown**	
	“Unable to go to school. Greatly misses social interaction with others. Not exposed to new ideas and activities. Is bored.”	
	“The major problems are that he cannot go to kindergarten and experience things with other children and that he cannot do proper therapy but only some exercise with power points.”	
	“Anxiety, restlessness, unknown Daily routine, lack of motivation, lack of stimulation, lack of education/homework/projects to work with therefore boredom.”	
	“Frustration at lack of routine. Disinterest and shortened attention span for former “school” tasks. Missing social interactions with teachers and peers.”	

In 13% of cases, relatives did not comment on medical problems that occurred during the lockdown. The absence of medical problems was reported in 63% of cases. In the remaining 24%, the main medical problems were digestive problems (6%), mental health problems (4%), skin problems (3%), epilepsy (3%), non-COVID-19 infectious disease (2.5%) and musculoskeletal problems (2.5%). Only 1% of the participants were suspected of having COVID-19.

## Discussion

This study investigated the effect of lockdown in the vulnerable population with genetic NDDs, including ID and ASD. First, our results highlight that this vulnerability is enhanced by the presence of behavioural problems and an ASD diagnosis prior to lockdown. These problems became more intense during lockdown with a greater tendency to depressiveness, aggressivity, self-aggressivity, stereotypes and restricted interests. Secondly, the sociability was impaired during lockdown with less social interactions with familiar children and unfamiliar people. And finally, our results showed a same amount of daily living difficulties during lockdown compared to the pre-lockdown period.

To start with the latter, no impact of the lockdown on the frequency of behavioural, eating and sleep problems was evidenced in this study. This finding is consistent with a study conducted in the United Kingdom showing that the well-being of families of children with ID (as reported by relatives) was at similar levels as before the lockdown period [31]. Thus, our results did not confirm predictions of a potential negative effect of lockdown on people with ID, but it does represent an additional burden in the lives of people with ID who already face a number of challenges daily. The impact could in fact be very heterogeneous with a positive, negative, or no effect on daily life as illustrated by the diversity of relatives’ comments, as had already been reported in a qualitative study in young children with intellectual and developmental disabilities [32]. The aim would then be to identify risk factors or determinants impeding the evolution of functional adaptation of people with ID during lockdown, to prevent negative consequences. Interestingly, we have shown that the intensity of behavioural problems only increased during lockdown for those who had such problems before. The individuals are therefore at risk for an increase in existing symptoms, as suspected by other authors [1]. All these results suggest that negative impact of lockdown is not related to ID per se but to the associated comorbidities such as behavioural problems. From the point of prevention, it is therefore recommended to pay extra attention to those who already experience behavioural problems.

Individuals with behavioural problems in both periods exhibited more severe disorders during lockdown, including more pronounced aggressivity, self-aggressivity, depressiveness, stereotypies, and restricted interests than in the pre-lockdown period. These results echoed past studies that showed higher behavioural problems during lockdown in developmental and ID [10, 33, 34], Down Syndrome [12], Prader-Willi Syndrome [22], Fragile X [21] and Dravet Syndrome [35]. Depression in people with ID often presents atypically, with agitation and irritability rather than a sad mood [36, 37]. From this perspective, the increased intensity of depression and aggression may be related to each other.

Besides mood problems, stereotypies also tend to worsen during lockdown. Sensory seeking is an intrinsic motivation for stereotypies and repetitive behaviours in ID [38]. In addition, stereotypies have been described as a form of sensory self-stimulation or automatic reinforcement in which stimulation is intended to compensate for an external arousal deficit [39]. The aforementioned literature suggests that all the behavioural categories that worsen during lockdown, relate to stimulation dysfunction. This can include both a lack of stimulation (boredom in the new situation) and overstimulation by changing habitual routines. This resistance to change, also known as the need for "sameness" since Kanner's early descriptions [40], is consistent with the high prevalence of comorbid ASD or autistic features in ID. It is noteworthy that individuals with ASD have specific difficulties coping with change during lockdown. A Spanish study showed that children with ASD who did not maintain a routine during lockdown had higher average levels of anxiety than children who did maintain a routine [41]. A qualitative study emphasized the impact of new routines in 25 children with ASD (more than half had comorbid ID) [42]. A scoping review with thematic analysis on people with disabilities highlighted the psychological consequences of disrupting routines, activities, and support [33], particularly in people with ID [10]. Finally, this imbalance in stimulation could highlight the impact of quitting education and/or therapy [43], as underlined by the many comments of relatives (Table 6, part B) and qualitative studies in ASD [42] and in ID [44], but further research is needed to confirm this effect.

Based on relatives’ reports, the affected individuals had fewer social interactions (with both familiar and unfamiliar children, and with unfamiliar adults), except with respect to familiar adults with whom social relationships were spared. This alteration in sociability may only be due to the fact of staying at home, which intrinsically reduces social relationships for everyone. The decreased shyness observed during lockdown is probably due to these changes in the social environment. However, relationships with familiar children were also altered, and a diagnosis of comorbid ASD was associated with a greater impact on sociability. This latter finding supports other studies that showed the negative impact of lockdown in ID [34] and in ASD [45]. Thus, the alteration of the relationship with familiar children and the link between sociability and ASD suggest a specific effect of lockdown on individuals with ID beyond the simple consequences of having to stay at home with their family. Further research is needed to test this assumption by comparing unaffected people with those with NDDs.

Our study is based on a high number of participants, thus involving many countries and genetic defects. Moreover, despite the declarative nature of these online collected data, the studied population is well characterized. Based on relatives’ declarations, half of the affected individuals with a comorbid ASD diagnosis were assessed using standard and required tools. The IQ test results reported by relatives corresponded to the level of reported ID level. Our study seems very ecological because it is based on the relatives' declarations in the daily environment independently of the intervention of professionals. Finally, by identifying possible risk factors related to lockdown, our study addresses a major prevention issue regarding people with ID given the probable future lockdowns.

We must stress some of the limitations of our study. The behavioural, eating and sleep problems were not assessed with standardized and validated tools. Therefore, we did not talk about “disorders” and “depression”, for example, but about “problems” and “depressiveness.” Hence, it would be very difficult to find tests that could adapt to the heterogeneity of age and level of ID in the population studied. Moreover, the data are based on opinions of relatives. As far as possible, we would like to strengthen our quantitative results with qualitative data based on interviews with the individuals with ID themselves as recommended by Gilson et al*.* [46]. However, such interviews were not adapted to the online framework of this study. Due to ethical restrictions, socio-economic status was not taken into account although studies have highlighted the impact of this status on people during lockdown [47, 48]. Finally, looking at the population studied, there is a bias towards certain genetic conditions that are overrepresented in the study population. The most represented genetic conditions were Koolen-de Vries syndrome (*n* = 49), Kleefstra syndrome (*n* = 26) and DDX3X-associated disorder (*n* = 11) (Supplementary Fig. 1).

## Conclusions

Our results support previous studies that showed that the negative impact of lockdown does not depend on the ID per se but on the associated comorbidities such as behavioural disorders. For individuals with NDDs and behavioural problems COVID-19 related restrictions worsened behavioural problems with more aggressivity, self-aggressivity, depressiveness, stereotypes and restricted interests. Finally, our study addresses a major prevention issue regarding vulnerabilities in people with genetic NDDs.

## Supplementary Information

**Additional file 1.** 

**Additional file 2:** **Fig S1.** Genetic defects or genes implicated in the ID of the participants to the study

**Additional file 3:** **Fig S2. **Participants’ countries of origin

**Additional file 4:** **Table S1. **Interaction between behaviour problems (beforeand during lockdown) and the factors studied

**Additional file 5:** **Table S2. **Interaction betweensociability (before and during lockdown) and the factors studied

## Data Availability

The datasets generated and/or analysed during the current study are not publicly available because they contain information that could compromise research participant privacy/consent but are available from the corresponding author on reasonable request.

## Abbreviations

ADHD: Attention-Deficit Hyperactivity Disorder
ADI-R: Autism Diagnostic Interview-Revised
ADOS: Autism Diagnostic Observation Schedule
ANOVA: ANalysis Of Variance
ASD: Autism Spectrum Disorder
ID: Intellectual Deficiency
IQ: Intellectual Quotient
KdVS: Koolen-de Vries syndrome
NDD: Neurodevelopmental disorders
OCD: Obsessive–Compulsive Disorder
PCA: Principal Component Analysis
