# Supplementary material for: The impact of lockdown on young people with genetic neurodevelopmental disabilities: a study with the international participatory database GenIDA
Source: BMC Psychiatry. 2022 Aug 25;22:572. doi: 10.1186/s12888-022-04213-6 (PMC9403223; doi:10.1186/s12888-022-04213-6)
Supplement: Supplementary file 1 — Additional file 1. [file 12888_2022_4213_MOESM1_ESM.docx]

**Additional file 1:** Lockdown questionnaire published online on the GenIDA platform

**Lockdown questionnaire**

*Evaluating the impact of lockdown/confinement linked to Covid-19 outbreak on the behaviour of people with intellectual disabilities or autism spectrum disorders*

**Section 1 - Diagnostics of intellectual disability & autism**

**1/15.** Diagnosis of intellectual disability?

(Unique choice)

- Yes
- No
- I don't know

What is the degree of this intellectual disability?

- Mild
- Moderate
- Severe
- Profound

**2/15.** Has an IQ test been performed?

(Unique choice)

- Yes
- No
- I don't know

If ‘yes’ ticked:

- Date the test was performed:

- Which test? *free text*

**3/15.** Diagnosis of autism? (Autism Spectrum Disorder)

(Unique choice)

- Yes
- No
- I don't know

**4/15.** Has an Autism test been performed?

(Unique choice)

- Yes
- No
- I don't know

If ‘yes’ ticked:

- Date the test was performed:

- Which test? *free text*

- Test result: *numerical value*

**Section 2 - Situation during the 6 months period BEFORE lockdown**

**5/15.** Behaviour problems BEFORE lockdown/confinement:

(Unique choice)

- Yes
- No
- I don't know

What words best describe them?

|  | Nonexistent | Mild | Moderate | Major |
| --- | --- | --- | --- | --- |
| Aggressivity |  |  |  |  |
| Self-aggressivity (self-mutilation) |  |  |  |  |
| Impulsivity |  |  |  |  |
| Hyperactivity |  |  |  |  |
| Attention deficit |  |  |  |  |
| Shyness |  |  |  |  |
| Anxiousness |  |  |  |  |
| Depressive tendencies |  |  |  |  |
| Restricted interests |  |  |  |  |
| Repetitive behaviour / stereotypes |  |  |  |  |
| Obsessions |  |  |  |  |
| Phobias |  |  |  |  |
| Problems related to a former diagnosis of schizophrenia |  |  |  |  |

Other? (specify) *free text*

**6/15.** Feeding problems BEFORE lockdown/confinement:

(Unique choice)

- Yes
- No
- I don't know

What words best describe them?

- Eats too much
- Craves for food
- Eats only very restricted food
- Does not want to eat (anorexia)
- Other: (specify) *free text*

How do you evaluate its (or their) seriousness?

- Major
- Moderate
- Mild

**7/15.** How would you describe your relative's sociability BEFORE lockdown/confinement?

Age (mandatory)

In each of these four cases:

|  | Very sociable | Average sociability | Little sociability | No interaction |
| --- | --- | --- | --- | --- |
| With familiar adults |  |  |  |  |
| With unfamiliar adults |  |  |  |  |
| With familiar children |  |  |  |  |
| With unfamiliar children |  |  |  |  |

**8/15.** Sleeping disorders BEFORE lockdown/confinement:

(Unique choice)

- Yes
- No
- I don't know

What words best describe them?

- Sleep apnea
- Other: (specify) *free text*

How do you evaluate its (or their) seriousness?

- Major
- Moderate
- Mild

**Section 3 - Situation UNDER lockdown**

**9/15.** Lockdown/confinement situation

The affected individual lives

- Within the family
- Outside the family

Housing condition

- Apartment
- Family home
- Other: (specify) *free text*

At home, the affected individual has

- A shared bedroom
- An own bedroom

Easy access to gardens or parks

- Yes
- No

Duration of the confinement at present: (in days)

**10/15.** Behavior problems UNDER lockdown/confinement:

(Unique choice)

- Yes
- No
- I don't know

What words best describe them?

|  | Nonexistent | Mild | Moderate | Major |
| --- | --- | --- | --- | --- |
| Aggressivity |  |  |  |  |
| Self-aggressivity (self-mutilation) |  |  |  |  |
| Impulsivity |  |  |  |  |
| Hyperactivity |  |  |  |  |
| Attention deficit |  |  |  |  |
| Shyness |  |  |  |  |
| Anxiousness |  |  |  |  |
| Depressive tendencies |  |  |  |  |
| Restricted interests |  |  |  |  |
| Repetitive behaviour / stereotypes |  |  |  |  |
| Obsessions |  |  |  |  |
| Phobias |  |  |  |  |
| Problems related to a former diagnosis of schizophrenia |  |  |  |  |

Other? (specify) *free text*

**11/15.** Feeding problems UNDER lockdown/confinement:

(Unique choice)

- Yes
- No
- I don't know

What words best describe them?

- Eats too much
- Craves for food
- Eats only very restricted food
- Does not want to eat (anorexia)
- Other: (specify) *free text*

How do you evaluate its (or their) seriousness?

- Major
- Moderate
- Mild

**12/15.** How would you describe your relative's sociability UNDER lockdown/confinement?

Age (mandatory)

In each of these four cases:

|  | Very sociable | Average sociability | Little sociability | No interaction |
| --- | --- | --- | --- | --- |
| With familiar adults |  |  |  |  |
| With unfamiliar adults |  |  |  |  |
| With familiar children |  |  |  |  |
| With unfamiliar children |  |  |  |  |

**13/15.** Sleeping disorders UNDER lockdown/confinement:

(Unique choice)

- Yes
- No
- I don't know

What words best describe them?

- Sleep apnea
- Other: (specify) *free text*

How do you evaluate its (or their) seriousness?

- Major
- Moderate
- Mild

**14/15.** What are the major problems that affected the everyday life (quality of life) of the affected individual UNDER lockdown/confinement?

*Free answer*

**15/15.** What are the major medical problems that occurred UNDER lockdown/confinement?

*Free answer*
