# Supplementary material for: The impact of lockdown on young people with genetic neurodevelopmental disabilities: a study with the international participatory database GenIDA
Source: BMC Psychiatry. 2022 Aug 25;22:572. doi: 10.1186/s12888-022-04213-6 (PMC9403223; doi:10.1186/s12888-022-04213-6)
Supplement: Supplementary file 2 — Additional file 2: Fig S1. Genetic defects or genes implicated in the ID of the participants to the study [file 12888_2022_4213_MOESM2_ESM.docx]

**Fig S1:** Genetic defects or genes implicated in the ID of the participants to the study
