# Supplementary material for: The impact of lockdown on young people with genetic neurodevelopmental disabilities: a study with the international participatory database GenIDA
Source: BMC Psychiatry. 2022 Aug 25;22:572. doi: 10.1186/s12888-022-04213-6 (PMC9403223; doi:10.1186/s12888-022-04213-6)
Supplement: Supplementary file 3 — Additional file 3: Fig S2. Participants’ countries of origin [file 12888_2022_4213_MOESM3_ESM.docx]

**Fig S2:** Participants’ countries of origin
