# Supplementary material for: The impact of lockdown on young people with genetic neurodevelopmental disabilities: a study with the international participatory database GenIDA
Source: BMC Psychiatry. 2022 Aug 25;22:572. doi: 10.1186/s12888-022-04213-6 (PMC9403223; doi:10.1186/s12888-022-04213-6)
Supplement: Supplementary file 4 — Additional file 4: Table S1. Interaction between behaviour problems (beforeand during lockdown) and the factors studied [file 12888_2022_4213_MOESM4_ESM.docx]

**Table S1:** Interaction between behaviour problems (before and during lockdown) and the factors studied

| **Cases** | **Sum of squares** | ***p*** |
| --- | --- | --- |
| Behaviour problems (before and during lockdown) (BPt1t2) | 22.357 | 0.116 |
| BPt1t2 ✻ Severity of ID | 0.245 | 0.868 |
| BPt1t2 ✻ Age | 0.143 | 0.899 |
| BPt1t2 ✻ ASD diagnosis | 0.327 | 0.848 |
| BPt1t2 ✻ Living with family | 16.858 | 0.172 |
| BPt1t2 ✻ House | 0.560 | 0.802 |
| BPt1t2 ✻ Sharing a bedroom | 4.440e-4 | 0.994 |
| BPt1t2 ✻ Easy access to park or garden | 0.444 | 0.823 |
| Residuals | 510.155 |  |
| *Note: Type III Sum of squares* | | |
