# Supplementary material for: The impact of lockdown on young people with genetic neurodevelopmental disabilities: a study with the international participatory database GenIDA
Source: BMC Psychiatry. 2022 Aug 25;22:572. doi: 10.1186/s12888-022-04213-6 (PMC9403223; doi:10.1186/s12888-022-04213-6)
Supplement: Supplementary file 5 — Additional file 5: Table S2. Interaction betweensociability (before and during lockdown) and the factors studied [file 12888_2022_4213_MOESM5_ESM.docx]

**Table S2:** Interaction between sociability (before and during lockdown) and the factors studied

| **Cases** | **Sum of squares** | ***p*** |
| --- | --- | --- |
| Sociability before and during lockdown (SOCt1t2) | 0.038 | 0.954 |
| SOCt1t2 ✻ Age | 4.549e-4 | 0.995 |
| SOCt1t2 ✻ Severity of ID | 1.820 | 0.688 |
| SOCt1t2 ✻ ASD diagnosis | 43.115 | 0.053 |
| SOCt1t2 ✻ Living with family | 21.549 | 0.169 |
| SOCt1t2 ✻ House | 0.564 | 0.823 |
| SOCt1t2 ✻ Sharing a bedroom | 12.265 | 0.298 |
| SOCt1t2 ✻ Easy access to park or garden | 28.525 | 0.114 |
| Residuals | 1281.056 |  |
| *Note: Type III Sum of squares* | | |
